# Deciphering the role of QPCTL in glioma progression and cancer immunotherapy

**DOI:** 10.3389/fimmu.2023.1166377

**Published:** 2023-03-29

**Authors:** Yu’e Liu, Shaojuan Lu, Yihong Sun, Fei Wang, Shibo Yu, Xi Chen, Lei-lei Wu, Hui Yang, Yufeng Shi, Kaijun Zhao

**Affiliations:** ^1^ Department of Neurosurgery, Shanghai East Hospital, School of Medicine, Tongji University, Shanghai, China; ^2^ Shanghai Pudong Hospital, Pudong Medical Center, Fudan University, Shanghai, China; ^3^ Department of Pathology and Medical Biology, University of Groningen, University Medical Center Groningen, Groningen, Netherlands; ^4^ Department of Pediatrics, Children’s Nutrition Research Center, Baylor College of Medicine, Houston, TX, United States; ^5^ Department of Thoracic Surgery, Shanghai Pulmonary Hospital, School of Medicine, Tongji University, Shanghai, China; ^6^ Department of Neurosurgery, National Center for Neurological Disorders, Shanghai Key Laboratory of Brain Function Restoration and Neural Regeneration, Huashan Hospital, Fudan University, Shanghai, China; ^7^ State Key Laboratory of Medical Neurobiology and MOE Frontiers Center for Brain Science, Institute for Translational Brain Research, Fudan University, Shanghai, China; ^8^ Tongji University Cancer Center, Shanghai Tenth People's Hospital of Tongji University, Clinical Center for Brain and Spinal Cord Research, School of Medicine, Tongji University, Shanghai, China

**Keywords:** QPCTL, glioma, cancer immunotherapy, immune infiltration, biomarker

## Abstract

**Background:**

Glioma is the most lethal and most aggressive brain cancer, and currently there is no effective treatment. Cancer immunotherapy is an advanced therapy by manipulating immune cells to attack cancer cells and it has been studied a lot in glioma treatment. Targeting the immune checkpoint CD47 or blocking the CD47-SIRPα axis can effectively eliminate glioma cancer cells but also brings side effects such as anemia. Glutaminyl-peptide cyclotransferase-like protein (QPCTL) catalyzes the pyroglutamylation of CD47 and is crucial for the binding between CD47 and SIRPα. Further study found that loss of intracellular QPCTL limits chemokine function and reshapes myeloid infiltration to augment tumor immunity. However, the role of QPCTL in glioma and the relationship between its expression and clinical outcomes remains unclear. Deciphering the role of QPCTL in glioma will provide a promising therapy for glioma cancer immunotherapy.

**Methods:**

QPCTL expression in glioma tissues and normal adjacent tissues was primarily analyzed in The Cancer Genome Atlas (TCGA) database, and further validated in another independent cohort from the Gene Expression Omnibus (GEO) database, Chinese Glioma Genome Atlas (CGGA), and Human Protein Atlas (HPA). The relationships between QPCTL expression and clinicopathologic parameters and overall survival (OS) were assessed using multivariate methods and Kaplan-Meier survival curves. And the proteins network with which QPCTL interacted was built using the online STRING website. Meanwhile, we use Tumor Immune Estimation Resource (TIMER) and Gene Expression Profiling Interactive Analysis (GEPIA) databases to investigate the relationships between QPCTL expression and infiltrated immune cells and their corresponding gene marker sets. We analyzed the Differentially Expressed Genes (DEGs) including GO/KEGG and Gene Set Enrichment Analysis (GSEA) based on QPCTL-high and -low expression tumors.

**Results:**

In contrast to normal tissue, QPCTL expression was higher in glioma tumor tissue (*p* < 0.05). Higher QPCTL expression was closely associated with high-grade malignancy and advanced tumor stage. Univariate and multivariate analysis indicated the overall survival of glioma patients with higher QPCTL expression is shorter than those with lower QPCTL expression (*p* < 0.05). Glioma with QPCTL deficiency presented the paucity of infiltrated immune cells and their matching marker sets. Moreover, QPCTL is essential for glioma cell proliferation and tumor growth and is a positive correlation with glioma cell stemness.

**Conclusion:**

High QPCTL expression predicts high grades of gliomas and poor prognosis with impaired infiltration of adaptive immune cells in the tumor microenvironment as well as higher cancer stemness. Moreover, targeting QPCTL will be a promising immunotherapy in glioma cancer treatment.

## 1 Introduction

Malignant gliomas are the most common primary invasive brain tumors, accounting for approximately 25% of central nervous system tumors (1). WHO divided the gliomas into four grades: grade I and grade II are low-grade gliomas (LGGs) and grade III and grade IV are high-grade gliomas (2). The glioblastoma is the highest grade of glioma, with its mid-survival time within one and a half years after the operation. The common therapies for gliomas include radiotherapy, chemotherapy, and operation. However, therapeutic advances for glioblastoma have been minimal over the past two decades (3). The molecular biomarkers specific to tumor subsets and targeted therapies are promising and important research directions (4). Finding the biomarkers of glioma that can guide the prescription of a particular targeted therapy is necessary and imperative.

The tumor microenvironment (TME) is heterogeneous, mixed with the tumor, stroma, and endothelial cells. It is characterized by the cross-talk between tumor and the innate and adaptive immune cells (5). The immune cells interact with the tumor cells in the development and progression of gliomas (6, 7). The infiltration of immune cells into the TME and the intra-tumor landscape is crucial for cancer immunotherapy and cancer treatment (8–10). A lack of understanding of the cellular complexity and the molecular heterogeneity of immune infiltration in gliomas limit our steps in developing more effective immunotherapies for gliomas (11, 12).

Cancer immunotherapy manipulates immune cells to attack cancer cells by enhancing immune response, including immune checkpoint inhibition and adoptive immunotherapy. Immunotherapy in gliomas is developing fast. For example, the generation of glioma stem cell-specific CAR macrophages primes locoregional immunity for postoperative glioblastoma therapy (13). Targeting immune checkpoint CD47 *via* blocking the CD47-SIRPα pathway has been studied a lot in glioma (14, 15). The metabolic rewiring from glycolysis to fatty acid oxidation (FAO) upregulates the expression of CD47 by the acetylation of NF-kB/RelA, and correspondingly, the FAO promotes the glioma growth with CD47-mediated immune escape (16). To overcome the blood–brain barrier (BBB), a BBB-permeable nanocapsule has been designed to send the anti-CD47 antibody to the brain, and the phagocytosis of macrophages and microglia has been improved (17). The combination therapy RRX-001 (targeting CD47) and temozolomide (a clinical drug for GBM) is in phase I clinical trial (NCT02871843). Besides targeting CD47 in glioma cancer immunotherapy, the GD2-directed chimeric antigen receptor (CAR) T cells are also in phase I clinical trial (NCT04196413) (18). Therefore, cancer immunotherapy targeting glioma is developing fast. However, targeting CD47 may bring side effects like anemia as red blood cells will be removed when CD47 is deficient in their surfaces. The glutaminyl-peptide cyclotransferase-like protein (QPCTL) belongs to a family of enzymes that catalyze the formation of pyroglutamate (pGlu) at the N-terminus of proteins by converting glutamine into pGlu residue (19), and this post-translation modification is named pyroglutamylation. The pyroglutamylation of CD47 on tumor cells is critical for its binding to SIPRα on macrophages (20, 21). Therefore, QPCTL plays a key role in phagocytosis checkpoint immunotherapy (22). A recent study has found that QPCTL regulates macrophage and monocyte abundance and inflammatory signatures in the TME (23). Loss of QPCTL limits chemokine function and reshapes myeloid infiltration to augment tumor immunity (22). Microglia and monocytes are the effector cells of CD47-SIRPα antiphagocytic axis disruption against glioblastoma (24). All of the previous studies indicate that QPCTL has close relations with immune cell infiltration. However, its relations with gliomas require further investigation.

In this study, we have comprehensively explored the diversified role of QPCTL in the progression of gliomas for the first time. We checked the relationship between QPCTL and immune infiltration as well as glioma clinical outcome. Moreover, the DNA methylation status of QPCTL and the association between QPCTL DNA methylation and glioma prognosis were evaluated. The relationship between the QPCTL expression and the glioma stemness was assessed. Our findings shed light on the potential roles of QPCTL in gliomas.

## 2 Materials and methods

### 2.1 Data source

All the mRNA expression data of QPCTL and the matching clinical pathologic information were first checked at The Cancer Genome Atlas (TCGA) (https://genome-cancer.ucsc.edu/). The mRNA expression level was evaluated by transcripts per kilobase per million mapped reads (TPM). The Chinese Glioma Genome Atlas (CGGA) database (http://www.cgga.org.cn/index.jsp) contains clinical and sequencing data of over 2,000 brain tumor samples from Chinese cohorts and is equipped with a user-friendly web application for data storage and exploration (25). The expression of QPCTL level in CGGA was also explored.

### 2.2 The GEO database and the Human Protein Atlas

The GEO database is a comprehensive gene expression library in the National Center of Biotechnology Information (NCBI) (https://www.ncbi.nlm.nih.gov/geo/). The GSE45921 was downloaded from the GEO database and validated for survival analysis. Single-cell sequencing data are also from GEO. The Human Protein Atlas (HPA, https://www.proteinatlas.org/) offers a broad amount of proteomic and transcriptome information of distinct human samples, which consists of cell, tissue, and pathology atlases. Protein immunohistochemistry in normal human tissues and tumor tissues of QPCTL (antibody: HPA040797) was obtained from this online website, and the QPCTL protein level is much higher in the glioma group than in normal groups.

### 2.3 Survival and statistical analysis

To investigate whether QPCTL expression level affects the clinical outcomes of glioma patients, we divided the cancer samples into two groups with high and low expression according to the median mRNA expression value of QPCTL and then we constructed a prognostic classifier using Kaplan–Meier (KM) survival curves to compare the survival disparities (https://kmplot.com/analysis/).

### 2.4 Univariate and multivariate logistic regression analysis

The univariate Cox regression analysis was used for calculating the association between the expression level of QPCTL and the patient’s OS. Afterward, a multivariate analysis was used to assess if the QPCTL is an independent prognostic factor for glioma patient survival. QPCTL has statistical significance in Cox regression analysis when the *p*-value is less than 0.05.

### 2.5 Protein–protein interaction comprehensive analysis

Another online tool we used was the Search Tool for the Retrieval of Interacting Genes/Proteins (STRING) website (https://string-db.org/). The website hosts a big collection of integrated and consolidated protein–protein interaction data. After importing the QPCTL into the online tool STRING, we obtained the protein–protein interaction (PPI) network information. A confidence score > 0.7 was considered significant. Genemania (http://genemania.org/) is also a function-predicting website for interested genes. We explored the interaction network of QPCTL on this website too.

### 2.6 Immune infiltration analysis

To explore whether the expression of QPCTL is related to the tumor immune microenvironment, we studied the Tumor Immune Estimation Resource (TIMER). TIMER is a public website that covers 32 cancer types and encompasses 10,897 samples from the TCGA database, aiming to assess the abundance of immune inner infiltrates (http://cistrome.org/TIMER/). The correlation of QPCTL expression with the abundance of six types of infiltrating immune cells (CD8+ T cells, CD4+ T cells, B cells, dendritic cells, macrophages, and neutrophils) in glioma patients was evaluated *via* the TIMER database. The relationship between the expression of the QPCTL gene and the tumor purity was also displayed. The tumor immune infiltration analysis with more immune cells (total of 22) was analyzed *via* ssGSEA using the GSVA R package. Original data are from TCGA (https://portal.gdc.cancer.gov/).

### 2.7 Estimation of stromal and immune cells in malignant tumors using expression data

The Estimation of Stromal and Immune Cells in Malignant Tumors using Expression Data (ESTIMATE) is a package that uses gene expression data to predict the content of interstitial cells and immune cells in malignant tumor tissues (26). Based on the enrichment analysis of a single sample gene set (ssGSEA), the algorithm generates three scores: stromal score (recording the presence of stroma in tumor tissue), immune score (representing the infiltration of immune cells in tumor tissue), and estimated score (inferring tumor purity). The Stromal Score, Immune Score, and Estimate Score of QPCTL can be obtained at http://www.sangerbox.com/.

### 2.8 Gene correlation analysis

The Gene Expression Profiling Interactive Analysis (GEPIA) (http://gepia.cancer-pku.cn/index.html) is an online database that consists of 9,736 tumors and 8,587 normal samples from TCGA and GTEx data. It focuses on the analyses of the expression of RNA sequencing. Gene Classes and Isoform Classes exhibit the types of 60,498 genes and 198,619 isoforms. In the GEPIA database, the relation of QPCTL expression with multiple markers for immune cells was investigated. The *x*-axis was presented with the level of QPCTL expression, and the *y*-axis was plotted with other genes of interest. In addition, we used TIMER data to validate the genes that were of significant correlation with QPCTL expression in the GEPIA web.

### 2.9 Identification and enrichment analysis of DEGs

Distinct QPCTL subtype-related DEGs were identified using the “limma” package in R (adj.*p* < 0.05 and |log_2_FC| > 2) (27). The functional and enrichment pathways (GO and KEGG) of DEGs were further explored using the “cluster profiler” package in R (28).

### 2.10 Function and pathway analysis by gene set enrichment analysis

Differentially expressed genes (DEGs) between low- and high-QPCTL expression groups were identified using the DESeq 2 R package. In this study, GSEA was performed using the ggplot2 R package to demonstrate the significant functions and pathways between the two groups. The expression level of QPCTL was used as a phenotype label. An adjusted *p*-value < 0.05, normalized enrichment score (|NES|) > 1.5, and false discovery rate (FDR) < 0.05 were considered a significant difference.

### 2.11 QPCTL gene essential analysis *via* CRISPR screen

To explore whether QPCTL is essential for glioma cells, we first checked BioGRID (https://thebiogrid.org/), which listed the CRISPR screen conducted in the literature. We also checked the cell viability when the QPCTL gene is knocked out from DepMap (https://depmap.org/).

### 2.12 Relation between QPCTL gene and immune checkpoints

From UCSC (https://xenabrowser.net/), we have downloaded a standardized pan cancer dataset: TCGA TARGET GTEx (PANCAN, N = 19,131, G = 60,499). Furthermore, we extracted the expression data of the marker genes of the ENS00000011478 (QPCTL) gene and two types of immune checkpoint pathway genes (*n* = 60) [Inhibitory (24), Stimulatory (29)] from the literature. The immune landscape of cancer in each sample was listed (30). Furthermore, we screened samples from various tumors. We also filtered all normal samples and further performed log2(*x*+0.001) transformation on each expression value. Next, we calculated the Pearson correlation between ENSG00000011478 (QPCTL) and the marker genes of five immune pathways. All the above can be realized from http://sangerbox.com/directly.

### 2.13 QPCTL expression in scRNA-seq

Tumor Immune Single-cell Hub 2 (TISCH2) is an scRNA-seq database, which aims to characterize TMEs at single-cell resolution. TISCH2 (http://tisch.comp-genomics.org) has collected 187 sets of high-quality tumor single-cell transcriptome data and corresponding patient information from GEO and ArrayExpress (31). The data cover 50 cancer types, including 6 million cells from more than 1,500 patients. Among them, 40 sets of TISCH2 data are single-cell transcriptome data under different treatment conditions, including immunotherapy, chemotherapy, targeted therapy, and combination therapy.

### 2.14 Relation between the expression of QPCTL and cancer immunotherapy

Tumor Immune Dysfunction and Exclusion (TIDE, website: http://tide.dfci.harvard.edu/) is a computational framework developed to evaluate the potential of tumor immune escape from the gene expression profiles of cancer samples. The highly scored genes in TIDE signatures also present potential regulators of tumor immune escape and resistance to cancer immunotherapies (32, 33).

### 2.15 Gene enrichment analysis

The highly expressed genes in QPCTL-high groups were subjected to Gene Ontology (GO) and Kyoto Encyclopedia of Genes and Genomes (KEGG) analysis at https://metascape.org.

### 2.16 The combination analysis of GO/KEGG and LogFC

We checked the relations between the expression of QPCTL and the biological process (BP), cellular component (CC), and molecular function (MF) *via* GO analysis; then, we conducted KEGG pathway analysis. On the basis of the GO and KEGG analyses, with the data of logFC, we calculated the *z* score of each item.

### 2.17 The DNA methylation level of QPCTL in glioma and normal tissue

The DNA methylation level of QPCTL in LGG and GBM was explored from DiseaseMeth **(** http://bio-bigdata.hubmu.edu.cn/diseasemeth/). This dataset is focused on the methylation of genes in different cancers. The methylation of QPCTL in glioma and normal tissue was checked in DiseaseMeth and verified at https://mexpress.be/. The QPCTL DNA methylation level of Chinese glioma patients was checked at CGGA (http://www.cgga.org.cn/index.jsp).

### 2.18 The correlation between the DNA methylation level of QPCTL and the survival of glioma patients

The correlation between the DNA methylation level of QPCTL and the glioma patients’ survival was checked at https://ngdc.cncb.ac.cn/ewas/datahub, and for Chinese glioma patients, it was checked at the CGGA database (http://www.cgga.org.cn/index.jsp).

### 2.19 Relation between QPCTL gene and cancer stemness

We checked the Pearson correlation between the expression of QPCTL and cancer stemness in the pan-cancer atlas by http://sangerbox.com. The stemness was calculated based on the RNA-based stemness scores derived by the stemness group, the DNA methylation-based stemness scores derived by the stemness group, and other stemness probes (219 probes).

## 3 Results

### 3.1 Patient characteristics

We first checked the RNA-sequencing data and the clinical prognostic information of glioma from the TCGA database; 699 glioma samples and 5 normal samples were enrolled in the analysis. A total of 349 patients had a low expression of QPCTL and 350 patients had a higher expression of QPCTL. We summarized the clinical information and their relations with the expression of QPCTL in **Table 1**.

**Table 1:** The clinical characteristics of the glioma patients in the test and validation sets.

Characteristics	Low expression of QPCTL	High expression of QPCTL	*p*-value	Statistic	Method
*n*	349	350			
WHO grade, *n* (%)			8.13459E-38	170.8042165	Chisq test
G2	166 (26.1%)	58 (9.1%)			
G3	134 (21%)	111 (17.4%)			
G4	14 (2.2%)	154 (24.2%)			
IDH status, *n* (%)			1.55214E-41	182.2648408	Chisq test
WT	39 (5.7%)	207 (30%)			
Mut	308 (44.7%)	135 (19.6%)			
Age, *n* (%)			1.11484E-10	41.6089076	Chisq test
≤60	312 (44.6%)	244 (34.9%)			
>60	37 (5.3%)	106 (15.2%)			
Gender, *n* (%)			0.464038535	0.536135964	Chisq test
Female	144 (20.6%)	154 (22%)			
Male	205 (29.3%)	196 (28%)			
Histological type, *n* (%)			2.96141E-35	163.6679898	Chisq test
Glioblastoma	14 (2%)	154 (22%)			
Astrocytoma	106 (15.2%)	90 (12.9%)			
Oligoastrocytoma	89 (12.7%)	46 (6.6%)			
Oligodendroglioma	140 (20%)	60 (8.6%)			
Primary therapy outcome, *n* (%)			0.024957804	9.352113267	Chisq test
PD	57 (12.3%)	55 (11.8%)			
SD	100 (21.5%)	48 (10.3%)			
PR	43 (9.2%)	22 (4.7%)			
CR	93 (20%)	47 (10.1%)			
1p/19q codeletion, *n* (%)			1.36339E-21	91.10366307	Chisq test
Non-codel	208 (30.1%)	312 (45.1%)			
Codel	141 (20.4%)	31 (4.5%)			

### 3.2 QPCTL expression is higher in tumor samples than in normal tissues

**Figure 1:**
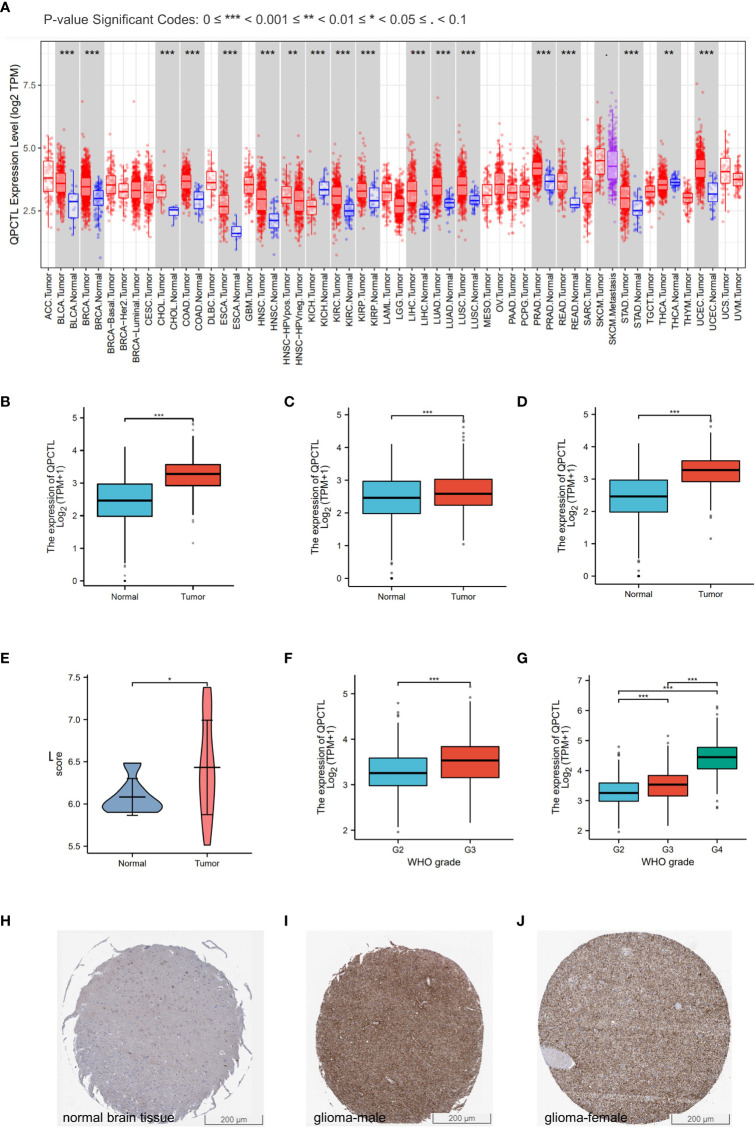
Higher QPCTL expression in tumor samples than that in normal tissues. **(A)** QPCTL expression at the mRNA level in pan-cancers, data from the TIMER database. **(B–D)** TCGA data show different QPCTL expression at mRNA levels in normal tissue and LGG **(B)**, GBMLGG **(C)**, and GBM **(D)**. **(E)** Different QPCTL expression in normal and low-grade glioma in the GEO database. **(F, G)** The expression of QPCTL in different stages of glioma in LGG **(F)** and GBMLGG **(G)**. **(H–J)** QPCTL protein expression in normal brain tissue and glioma tissue, data from the Human Protein Atlas. *p < 0.05; **p < 0.01; ***p < 0.001.

The mRNA expression of QPCTL in pan-cancer was browsed from the TIMER database (**Figure 1A**). QPTCL expression was significantly higher in tumor samples than in the normal tissues of LGG (**Figure 1B**), the combination of both GBM and LGG samples (GBMLGG) (**Figure 1C**), and glioblastoma multiforme (GBM) (**Figure 1D**) in TCGA, which was also validated in GEO database GSE45921 (**Figure 1E**). As shown in **Figure 1F** (LGG) and **Figure 1G** (GBMLGG), QPCTL expression level was observed in the higher tumor stage, as well as in higher grade and glioma classification, indicating that the expression of QPCTL is positively correlated with the glioma pathological stages. Since GBM is a grade IV (stage 4) glioma, the result in **Figure 1G** of G4 represents GBM data. Subsequently, the protein level of QPCTL was explored in the HPA, and it is shown that the QPCTL protein is higher in gliomas than in normal tissue (**Figures 1E–G**). Moreover, there is no significant difference between QPCTL expression and age and gender, but a higher expression of QPCTL indicates a higher grade of glioma. All the above results can also be validated in the CGGA (**Supplementary Figure 1**).

### 3.3 Higher QPCTL predicts shorter survival in glioma

**Figure 2:**
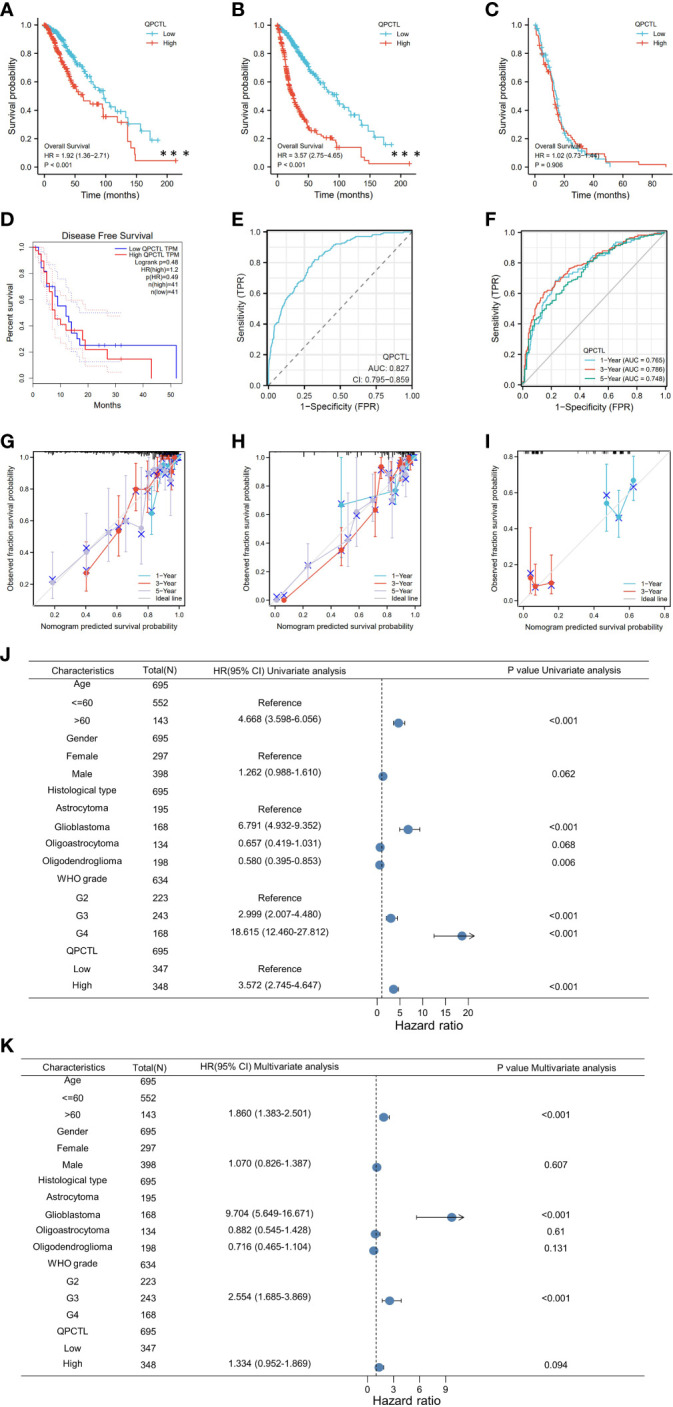
Higher QPCTL mRNA expression showing shorter OS in glioma. **(A–C)** The overall survival of QPCTL high and low expression of LGG patients **(A)**, GBMLGG **(B)**, and GBM **(C)** from the TCGA database. **(D)** The disease-free survival of low-grade glioma patients with high and low QPCTL expression. **(E)** The receiver operating characteristic curve (ROC) of QPCTL in GBM in TCGA. **(F)** The time-dependent ROC curve of GBMLGG. **(G–I)** The calibration curve of 1-, 3-, and 5-year survival of LGG patients **(G)**, GBMLGG **(H)**, and GBM **(I)** patients. **(J)** Univariate regression analysis of QPCTL and other clinicopathologic parameters with OS in LGG. **(K)** The multivariate regression analysis of QPCTL and other clinicopathologic parameters with OS in LGG.

As per the KM analysis of glioma patients’ survival, patients with higher QPCTL expression indicate a shorter overall survival (OS) in the test cohort in LGG (**Figure 2A**), GBMLGG (**Figure 2B**), and GBM (**Figure 2C**). The disease-free survival (DFS) of LGG patients from the GEPIA database also indicated that the higher expression of QPCTL was positively related to a shorter survival (**Figure 2D**). All the above results were found in CGGA data as well (**Supplementary Figure 2**).

We then checked whether QPCTL can be a predictor for clinical diagnosis since the evaluation of a risk prediction model is characterized in terms of discrimination and calibration. The receiver operating characteristic (ROC) curve, also named the sensitivity curve, is widely used for evaluating model discrimination and the performance of diagnostic tests. As shown in **Figure 2E**, the ROC curve indicates that QPCTL can be a prognostic marker of GBMLGG. The time-dependent ROC curve of GBMLGG is shown in **Figure 2F**. The calibration curve of 1-, 3-, and 5-year survival of LGG (**Figure 2G**) and GBM (**Figure 2H**) and 1- and 3-year survival of GBM (**Figure 2I**) is shown clearly. There are no data on the 5-year survival of GBM since it is a grade IV glioma and the mid-survival is 15 months. The coincidence between the actual incidence and the predicted incidence is the highest in the 5-year survival model. In the univariate Cox model, both higher QPCTL expression and high pathologic grade and stage were negative predictors for OS in glioma patients (**Figure 2J**). Intriguingly, in multivariate regression analysis, QPCTL expression was an independent factor corrected with OS in LGG patients (**Figure 2K**).

### 3.4 Differentiated gene analysis of QPCTL-high and -low groups in GBMLGG patients

**Figure 3:**
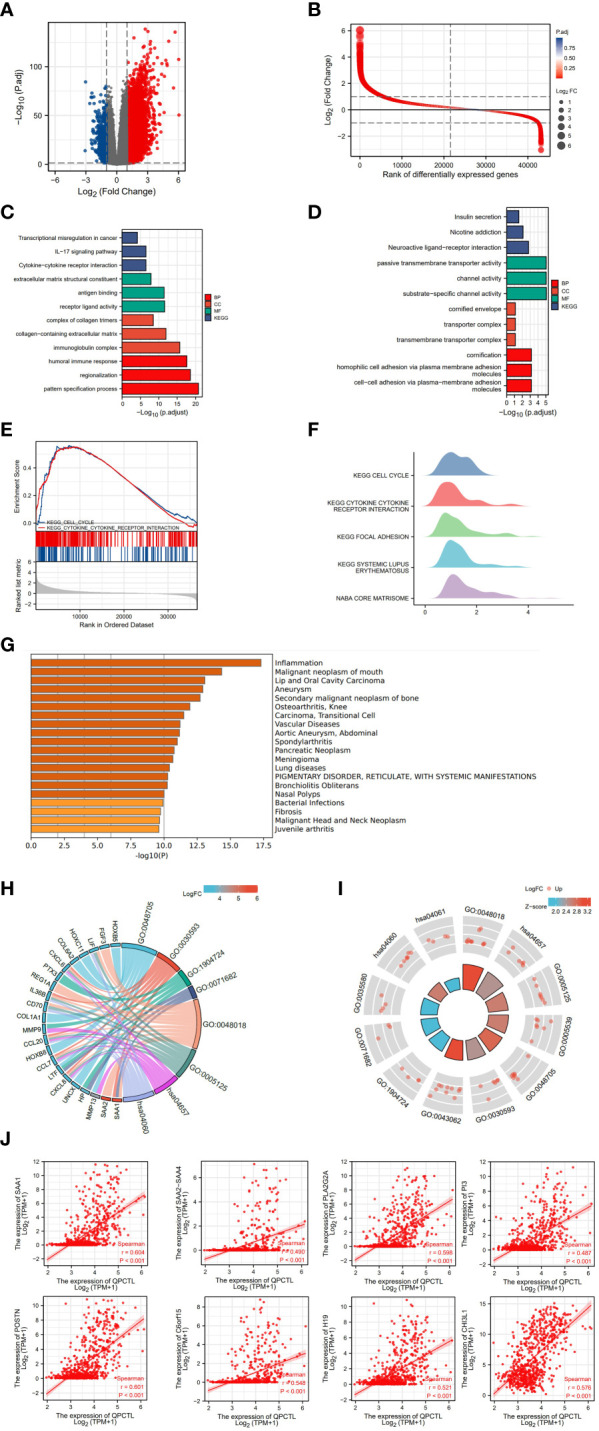
Differentiated gene analysis of QPCTL-high and -low groups in LGG patients. **(A)** The volcano plot shows the DEGs in QPCTL-high and -low groups. **(B)** The rank of differentially expressed genes. **(C)** The GO and KEGG analysis of high-expressed genes in QPCTL-high groups. **(D)** The GO and KEGG analysis of low-expressed genes in QPCTL-low groups. **(E)** The GSEA or the DEGs. **(F)** The GSEA in the ridge plot of DEGs. **(G)** The GO analysis of highly expressed genes *via* the website https://metascape.org/. **(H, I)** The combinational analysis of GO and KEGG of high-expressed genes in the QPCTL-high group. **(J)** The Pearson correlations between QPCTL and the top eight highly expressed genes in DEGs.

From the survival analysis and prognosis modal, we found that QPCTL is a more suitable marker for GBMLGG. To explore the possible role of QPCTL in the carcinogenesis of GBMLGG, we divided the patient samples according to QPCTL expression, and the DEG analysis was conducted in QPCTL-high and QPCTL-low groups. The volcano plot in **Figure 3A** shows the DEGs in both groups, and **Figure 3B** shows the rank of DEGs. To check how these DEGs are functioning in the biological pathways and cellular functions, we next performed GO and KEGG analysis; as shown in **Figure 3C**, the high-expressed genes in QPCTL-high groups are enriched in IL-17 signal pathway and cytokine–cytokine receptor interaction. The low-expressed genes in QPCTL-low groups are enriched in nicotine addiction and neuroactive ligand–receptor interaction (**Figure 3D**). The gene set enrichment analysis (GSEA) shows the high-expressed genes enriched in cytokine–receptor interaction and cell cycle pathway (**Figures 3E, F**). We verified the analysis at Metascape and found that the enrichment analysis was the highest enriched in the inflammation pathway (**Figure 3G**). The combinational analysis of GO/KEGG and logFC was also conducted, as shown in **Figures 3H, I**; the key pathways and involved genes are shown in the figures. Most of them are related to cytokines or other immune function types. We thus checked the relation between QPCTL and the top 8 expressed genes with fold change more than 4 times and found that the expression of QPCTL was positively correlated with their expression (**Figure 3J**).

### 3.5 Constructing protein interaction networks

**Figure 4:**
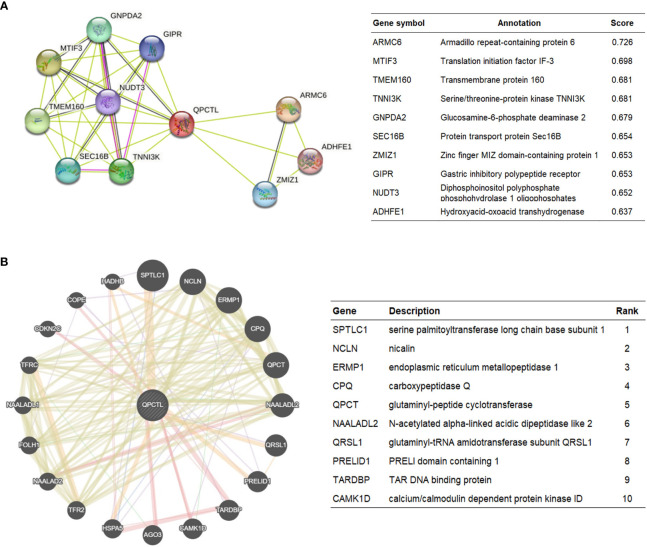
QPCTL–interaction proteins. **(A)** Annotation of QPCTL-interacting proteins and their co-expression scores, data from STRING website (https://string-db.org/). **(B)** Annotation of QPCT–interaction proteins and their correlation scores, data from Geneaminia website (http://genemania.org/).

The protein interaction network is necessary for the molecular mechanism of malignancy. Therefore, we used the STRING website to analyze the PPI network of the QPCTL protein to determine their interactions in the progression of glioma. The top 10 proteins and corresponding gene names, annotations, and scores are listed in **Figure 4A**. We also checked the protein interaction in Geneamia and obtained the network as shown in **Figure 4B**. The interaction proteins are different from the two other websites, probably due to their different calculation and original database. All the PPI networks explored on the two websites are quite different from our previous analysis; to focus on our study, we used our previous GO and KEGG analysis results to conduct the succeeding analysis; thus, we will focus on the expression of QPCTL and cytokines in the immune cells and the immune cell infiltration in gliomas.

### 3.6 Correlation analysis between QPCTL expression and infiltrating immune cells

**Figure 5:**
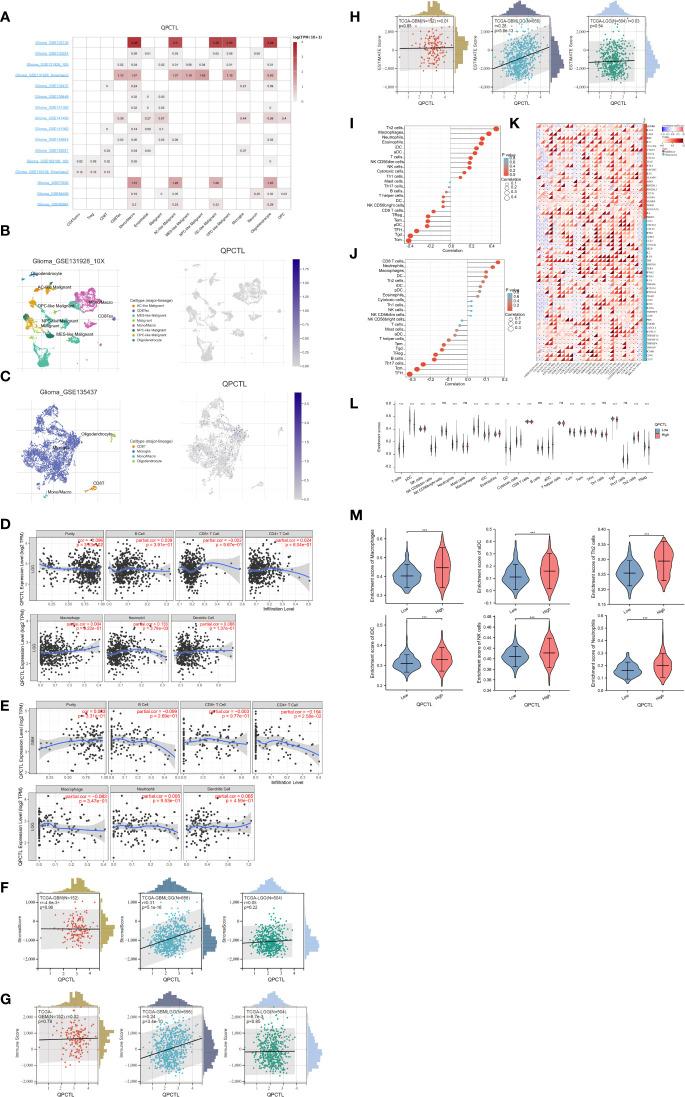
Correlation analysis between QPCTL expression and infiltrating immune cells **(A)** The expression of QPCTL in different immune cells, data from GEO data single-cell sequencing. **(B)** QPCTL expression in the glioma single-cell sequencing data (GSE131928). **(C)** QPCTL expression in the glioma single-cell sequencing data (GSE135437). **(D)** Correlation of QPCTL expression with infiltrating immune infiltration in LGG from TIMER database. **(E)** Correlation of QPCTL expression with infiltrating immune infiltration in GBM from the TIMER database. **(F)** Relation between QPCTL expression and stromal score in GBM, GBMLGG, and LGG. **(G)** Correlation of QPCTL expression with infiltrating immune score in GBM, GBMLGG, and LGG from the TCGA database. **(H)** Correlation of QPCTL expression with ESTIMATE score in GBM, GBMLGG, and LGG from the TCGA database. **(I)** The immune cell infiltration in the QPCTL-high and -low groups. **(J)** The immune cells with higher infiltration in QPCTL-high groups. **(K)** The correlation between the expression of QPCTL and the immune checkpoints. **(L)** The enrichment scores of immune cells in QPCTL-high and -low group. **(M)** The enrichment score of macrophage, iDC (immature DC cell), aDC (activated DC cell), Th2 cell, and Neutrophils in the QPCTL-high and -low groups. Data from http://sangerbox.com/, TCGA TARGET GTEx (PANCAN, N = 19,131, G = 60,499). **p < 0.01, ***p < 0.001.

We have a general view of the expression of QPCTL in all different immune cells, as shown in **Figure 5A**; the single-cell sequencing data from GEO indicate that QPCTL expresses higher in monocyte/macrophages. Single-cell sequencing of GSE131928 (**Figure 5B**) and GSE135437 (**Figure 5C**) shows that QPCTL expresses most in mono/macroglia and microglia, respectively.

Tumor-infiltrating lymphocytes are closely associated with the survival of patients with various cancers. Therefore, we analyzed the correlation of QPCTL expression with six kinds of infiltrating immune cells, namely, CD8^+^ T cells, CD4^+^ T cells, B cells, dendritic cells, macrophages, and neutrophils, and tumor purity from the TIMER website. The results showed that the expression level of QPCTL has no obvious correlation with the infiltration levels of these six immune cells in GBMLGG (**Figure 5D**) and in GBM (**Figure 5E**).

Infiltrating stromal and immune cells form the major fraction of normal cells in tumor tissue and not only perturb the tumor signal in molecular studies but also have an important role in cancer biology (26). They are important factors in the TME. Therefore, we also use ESTIMATE data to check the Stromal Score (**Figure 5F**), the Immune Score (**Figure 5G**), and the Estimate Score (**Figure 5H**) of QPCTL in LGG, GBMLGG, and GBM. The relations between the expression and the above scores indicate that the expression of QPCTL was positively related to the immune infiltration in GBMLGG instead of LGG or GBM. We also explored more immune cell infiltration (a total of 22 immune cells) in **Figure 5I** for GBMLGG and **Figure 5J** for GBM *via* ssGSEA. It shows that the expression of QPCTL in GBMLGG was positively correlated with Th2 cells and macrophages, while it was positively correlated with CD8^+^ T cells and neutrophils in GBM.

QPCTL as an immune checkpoint regulator has been verified as it is a critical enzyme to catalyze the pyroglutamylation of CD47 (20, 34). High expression of checkpoints suppresses the immune response and reduces the efficacy of immunotherapy; we then explore the relationship between the expression of QPCTL and the expression of checkpoint genes in pan-cancer. As shown in **Figure 5K**, the expression of QPCTL has positive relations with many immune checkpoints. In addition, to explore the relationship between the expression of QPCTL and immune cell infiltration, we checked the infiltration of the immune cells in the QPCTL-high and -low groups (**Figure 5L**) and found that the innate immune cell infiltration was more than the adaptive immune cell infiltration in the QPCTL-high groups (**Figure 5M**).

### 3.7 The DNA methylation status of QPCTL and its correlation with glioma patients’ survival

**Figure 6:**
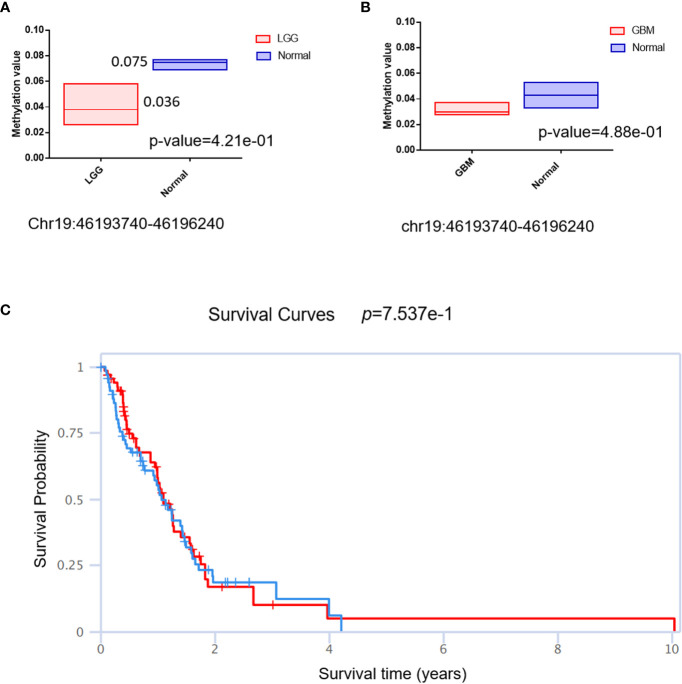
The DNA methylation status of QPCTL and its relationship with the glioma patients’ survival. **(A)** The DNA methylation level of QPCTL in LGG patients and normal people. **(B)** The DNA methylation level of QPCTL in GBM patients and normal people. **(C)** The correlation between the DNA methylation of QPCTL and the GBM patients’ survival.

The DNA methylation status of the key gene has been widely used as a biomarker in cancer; we then checked the methylation status of QPCTL in gliomas. As shown in **Figure 6A** (LGG) and **Figure 6B** (GBM), the DNA methylation of QPCTL promoters in glioma patients is much lower than that in the normal samples. This result is consistent with the mRNA expression of QPCTL in glioma patients since the lower DNA methylation indicates higher gene expression. The DNA methylation level of QPCTL becomes much lower from grade II to grade IV (**Supplementary Figure 3**), indicating that the DNA methylation of QPCTL predicts the pathological process of glioma. We then checked the correlation between the DNA methylation of QPCTL and the glioma patients’ survival and found higher methylation level of QPCTL had a longer survival in GBM (**Figure 6C**, **Supplementary Figure 4**), but it varies in different stages of gliomas (**Supplementary Figure 4**). In summary, the DNA methylation of QPCTL is negatively related to its expression and positively related to glioma patients’ survival.

### 3.8 QPCTL is essential for glioma cell proliferation and positively correlated with glioma stemness

**Figure 7:**
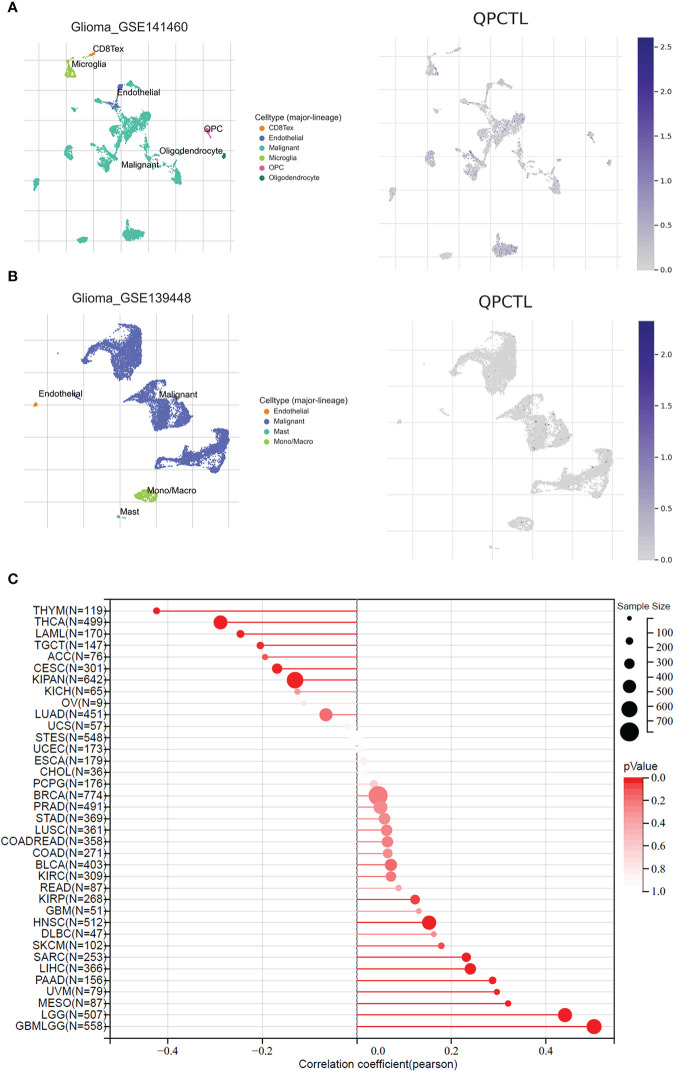
QPCTL is essential for glioma cell proliferation and positively correlated with glioma stemness. **(A)** The expression of QPCTL in GSE 141460, data from GEO single-cell sequencing. **(B)** The expression of QPCTL in GSE 139448, data from GEO single-cell sequencing. **(C)** The correlation between the expression of QPCTL and the cancer stemness, data from TCGA.

To explore the importance of QPCTL in glioma cells, we checked both the DepMap database and the BioGRID database, as shown in **Supplementary Table 1** (data from BioGRID) and **Supplementary Table 2** (data from DepMap); both CRISPR databases show that QPCTL is the essential gene for glioma cells’ proliferation. Among the 74 glioma cells subjected to CRISPR whole-genome screening, 63 cells’ proliferation was seriously suppressed (**Supplementary Table 1**), indicating that QPCTL was essential for glioma cells’ proliferation and tumor growth. Mechanistically, knocking out QPCTL attenuated tumor growth *via* disrupting monocyte homeostasis (22). As shown in **Figures 7A, B**, based on single-cell sequencing data from GEO (GSE 141460, GSE 139448), the expression of QPCTL is higher in the malignant glioma clusters than in other clusters, indicating that the expression of QPCTL has close positive relations with the glioma tumor progression.

Cancer stem cells drive tumor initiation, expansion, and recurrence following chemotherapy (35), and higher cancer stem cells predict poor prognosis. Therefore, we explored the expression of QPCTL and the cancer stemness in pan-cancer including gliomas. As shown in **Figure 7C**, the expression of QPCTL has a significant positive correlation with GBMLGG, and the correlation coefficient was the highest among all pan-cancers in TCGA, indicating that the QPCTL may also affect the glioma cancer stemness and promote tumor progression.

## 4 Discussion

In this study, we briefly explored the mRNA expression of QPCTL in gliomas and found that it was expressed higher in gliomas than in normal tissue, and the higher expression of QPCTL predicts a poorer prognosis of glioma. QPCTL can serve as an independent prognostic factor for OS. To further explore the function of QPCTL, we divided the gliomas into QPCTL-high and -low groups and then analyzed the DEGs. Interestingly, the GO/KEGG analysis of the QPCTL-high groups was enriched in the immunity-related pathways, especially the cytokine–cytokine receptor pathways. Since QPCTL expression is closely related to cytokine and chemokine function and modulation of QPCTL synergized with anti-PD-L1 expands CD8^+^ T cells and limits tumor growth, targeting QPTCL constitutes an effective approach for myeloid cell-targeted cancer immunotherapy; it prevents monocyte migration across inflammatory conditions (22). Knocking out QPCTL suppressed tumor growth *via* disrupting monocyte homeostasis, and knocking out QPCTL together with blocking immune checkpoint CD47 has been verified to improve the killing of T-acute lymphoblastic leukemia cells (29, 36). In a recent study, it was found that CD47 deficiency or pyroglutamylation inhibition increases myeloid cell-mediated T-ALL killing; QPCTL deficiency reduced 78% of the binding between CD47 and SIRPα (36). The regulation function of QPCTL of monocyte infiltration under inflammatory conditions had been demonstrated a long time ago (37). In renal cell carcinoma, the high expression of QPCTL leads to sunitinib resistance by promoting angiogenesis (38). In chronic kidney disease, chronic treatment with QPCTL inhibitor PQ529 is a novel and effective approach for glomerulonephritis (39). Aside from cancer, targeting QPCTL has been widely studied in Alzheimer’s disease as it modified the forms of amyloid-β (Aβ) oligomers to catalyze the generation of pyroglutamate-Aβ (AβpE3), which is more neurotoxic (21, 40). The small-molecule varoglutamstat (formerly PQ912) that inhibits the activity of QPCTL is currently in phase IIb clinical trials (41).

To further analyze the relationship between the expression of QPCTL and the TME, we conducted an immune microenvironment analysis and found that there was a significant difference in the proportion of immune cells between the QPCTL-high and -low expression groups. The infiltration level of different immune cells was significantly correlated with the expression of QPCTL. Our analysis indicates that the adaptive immune cell infiltration in the QPCTL-high groups was worse than that in the QPCTL-low groups. Furthermore, the expression of QPCTL was positively correlated with SAA1, POSTN, PLA2G2A, SAA2,C6orf15, H19, PI3, etc., whose expression also affects cancer progress. It has been found that the higher expression of SAA1 predicts advances and malignancies in various cancers (42–44). POSTN expression is crucial for the angiogenesis of gliomas (45–47).

The DNA methylation status of QPCTL and its relationship with glioma patients’ survival indicate that the DNA methylation status can be a predictor for the glioma pathological process. The highly positive correlation between QPCTL expression and glioma stemness also indicates that the expression of QPCTL has close relation with the progression of gliomas.

The advantage of our study is that it provides a novel biomarker of glioma, especially for GBMLGG, and we have explored the working mechanisms from different aspects. We found that the chemokine modulation role of QPCTL in the TME affects the immune responses. Owing to the specific role of QPCTL in the binding between CD47 and SIRPα, targeting QPCTL instead of targeting CD47 in gliomas overcomes the side effects of targeting CD47. It can be easily translated into an easy-to-use clinical assay to identify the efficacy of targeting QPCTL and the potential immunotherapy responders. The small-molecule inhibitors for targeting QPCTL to cure malignancies such as ISM004-1057D are in the pre-clinical stage now. However, there are still limitations in our research. For example, we did not verify the functions of QPCTL in all the gliomas by experiments; we just cited the previous CRISPR result. We will further study this gene and the deep mechanisms of glioma progression in the coming project.

## 5 Conclusion

In summary, our analysis revealed that the high expression of QPCTL is positively related to unfavorable outcomes in glioma. Further bioinformatic analysis indicates that QPCTL modulates the cytokine and cytokine–receptor pathway and thus affects the adaptive immune cells’ infiltration, which enhances the proliferation and progression of the cancer cells. Moreover, the DNA methylation status of QPCTL is closely associated with the patient’s survival. QPCTL is the essential gene for glioma cancer cells and its expression has a significantly positive correlation with glioma cancer stemness. Targeting QPCTL overcomes the side effects of targeting CD47; therefore, targeting QPCTL will be a promising strategy in glioma cancer treatment.

## Data availability statement

The original contributions presented in the study are included in the article/**Supplementary Material**. Further inquiries can be directed to the corresponding authors.

## Author contributions

KZ, YFS, and YL organized article writing and critically modified the manuscript. SL and YHS modified the manuscript. SY, XC, HY, and L-LW edited the manuscript and sourced literature. All authors read and approved the manuscript and agree to be accountable for all aspects of the research in ensuring that the accuracy or integrity of any part of the work is appropriately investigated and resolved.

## Supplementary material

Supplementary Figure 1 The mRNA expression of QPCTL in the CGGA database **(A)** The expression of QPCTL in different histology of glioma. **(B)** The expression of QPCTL in different grades of glioma. **(C-D)** The expression of QPCTL in IDH-mutant and wild-type gliomas. **(E-F)** the expression of QPCTL in different genders of glioma patients. **(G-H)** the expression of QPCTL in different ages of glioma patients. (LGG, lower-grade glioma; GBM, glioblastoma; A, astrocytoma; O, oligodendroglioma; OA, oligo‐astrocytoma; AOA, anaplastic oligo-astrocytoma; AA: anaplastic astrocytoma; rGBM: recurrent glioblastoma; rAA, recurrent anaplastic astrocytoma; rA, recurrent astrocytoma; AO, anaplastic oligodendroglioma; rAO, recurrent anaplastic oligodendroglioma; rO, recurrent oligodendroglioma; rAOA, recurrent anaplastic oligo‐astrocytoma.)

Supplementary Figure 2 The correlation between QPCTL expression and glioma patients’ survival in the CGGA database. **(A)** The correlation between the expression of QPCTL and the survival of primary glioma patients in all WHO grades. **(B)** The correlation between the expression of QPCTL and the survival of recurrent glioma patients in all WHO grades. **(C)** The correlation between the expression of QPCTL and the survival of primary glioma patients in grade II. **(D)** The correlation between the expression of QPCTL and the survival of recurrent glioma patients in grade II. **(E)** The correlation between the expression of QPCTL and the survival of primary glioma patients in grade III. **(F)** The correlation between the expression of QPCTL and the survival of recurrent glioma patients in grade III. **(G)** The correlation between the expression of QPCTL and the survival of primary glioma patients in grade IV. **(H)** The correlation between the expression of QPCTL and the survival of recurrent glioma patients in grade IV.

Supplementary Figure 3 The DNA methylation level of QPCTL in the CGGA database. **(A)** The DNA methylation level of QPCTL in different histology of glioma. **(B)** The DNA methylation level of QPCTL in different pathological stages of glioma. **(C)** The DNA methylation level of QPCTL in different genders. **(D)** The different DNA methylation levels of different genders in different pathological stages. **(E)** The DNA methylation level of QPCTL in different ages. **(F)** The different DNA methylation levels of different ages in different pathological stages.

Supplementary Table 1 QPCTL CRISPR screen data from BIOGRID.

Supplementary Table 2 QPCTL CRISPR screen data from DepMap.
