# Supplementary figures and images for: Deciphering the role of QPCTL in glioma progression and cancer immunotherapy
Source: Front Immunol. 2023 Mar 29;14:1166377. doi: 10.3389/fimmu.2023.1166377 (PMC10090505; doi:10.3389/fimmu.2023.1166377)

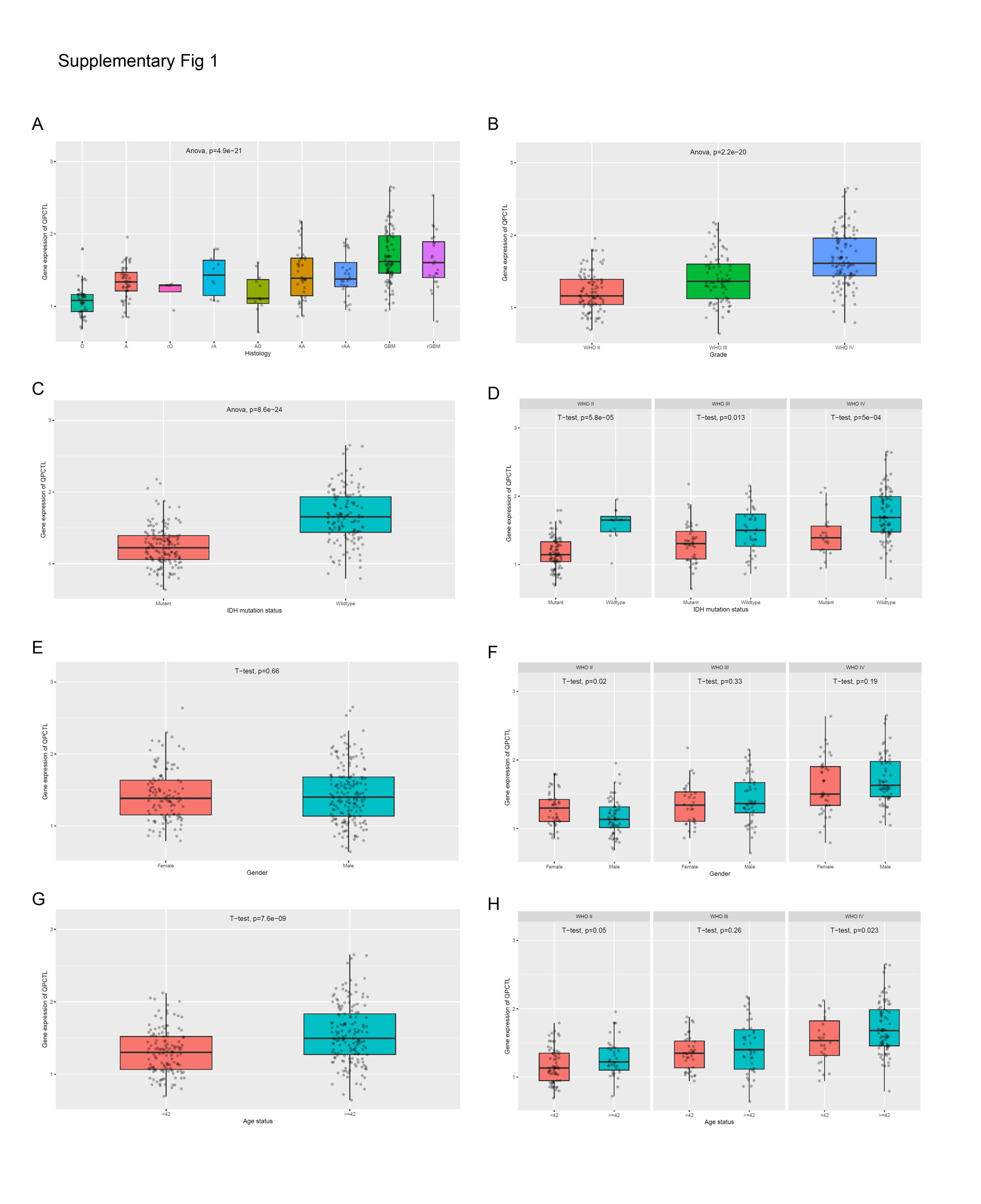

Supplement: Supplementary Figure 1 — The mRNA expression of QPCTL in the CGGA database (A) The expression of QPCTL in different histology of glioma. (B) The expression of QPCTL in different grades of glioma. (C-D) The expression of QPCTL in IDH-mutant and wild-type gliomas. (E-F) the expression of QPCTL in different genders of glioma patients. (G-H) the expression of QPCTL in different ages of glioma patients. (LGG, lower-grade glioma; GBM, glioblastoma; A, astrocytoma; O, oligodendroglioma; OA, oligo‐astrocytoma; AOA, anaplastic oligo-astrocytoma; AA: anaplastic astrocytoma; rGBM: recurrent glioblastoma; rAA, recurrent anaplastic astrocytoma; rA, recurrent astrocytoma; AO, anaplastic oligodendroglioma; rAO, recurrent anaplastic oligodendroglioma; rO, recurrent oligodendroglioma; rAOA, recurrent anaplastic oligo‐astrocytoma.) [file Image_1.jpeg]

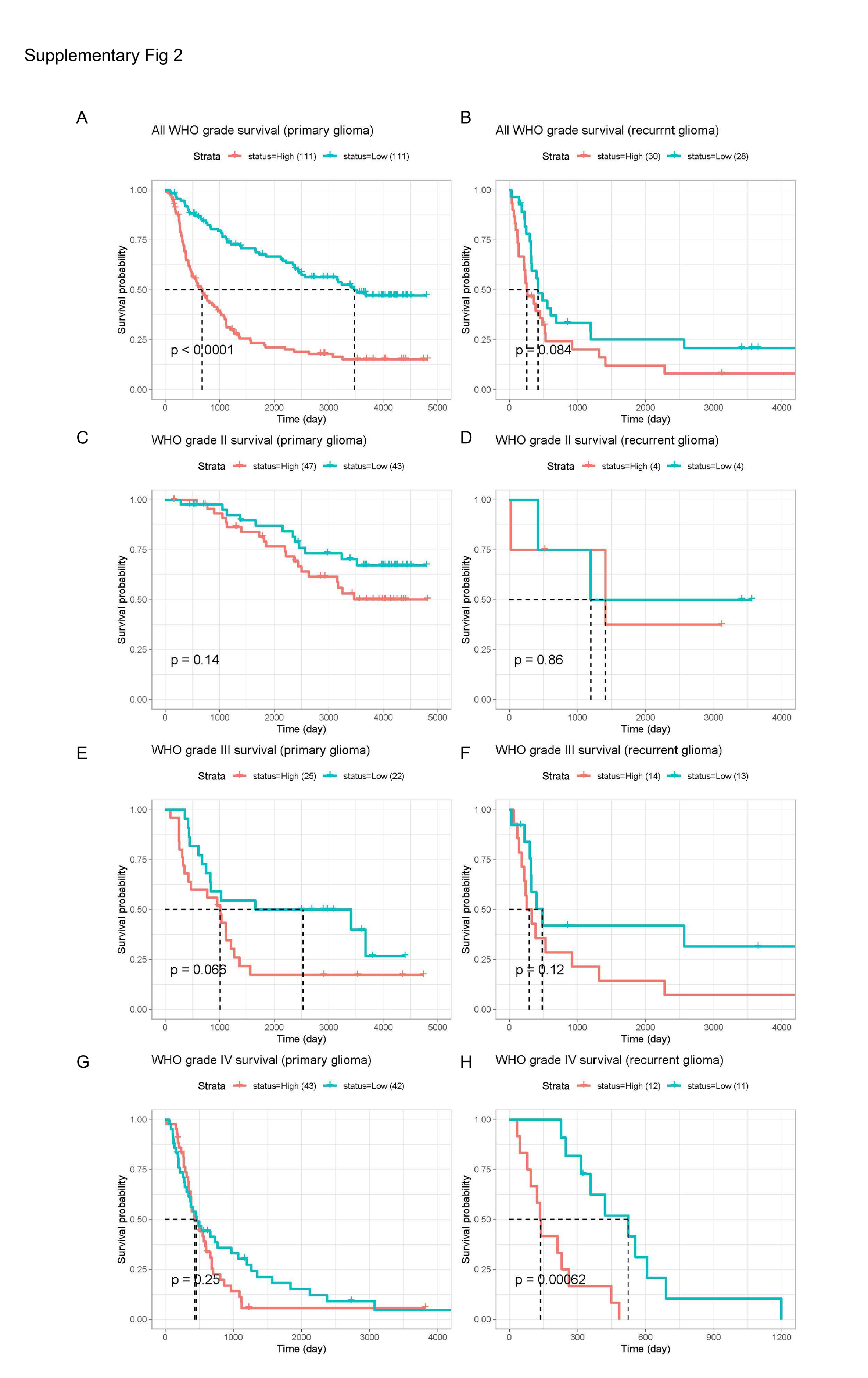

Supplement: Supplementary Figure 2 — The correlation between QPCTL expression and glioma patients’ survival in the CGGA database. (A) The correlation between the expression of QPCTL and the survival of primary glioma patients in all WHO grades. (B) The correlation between the expression of QPCTL and the survival of recurrent glioma patients in all WHO grades. (C) The correlation between the expression of QPCTL and the survival of primary glioma patients in grade II. (D) The correlation between the expression of QPCTL and the survival of recurrent glioma patients in grade II. (E) The correlation between the expression of QPCTL and the survival of primary glioma patients in grade III. (F) The correlation between the expression of QPCTL and the survival of recurrent glioma patients in grade III. (G) The correlation between the expression of QPCTL and the survival of primary glioma patients in grade IV. (H) The correlation between the expression of QPCTL and the survival of recurrent glioma patients in grade IV. [file Image_2.jpeg]

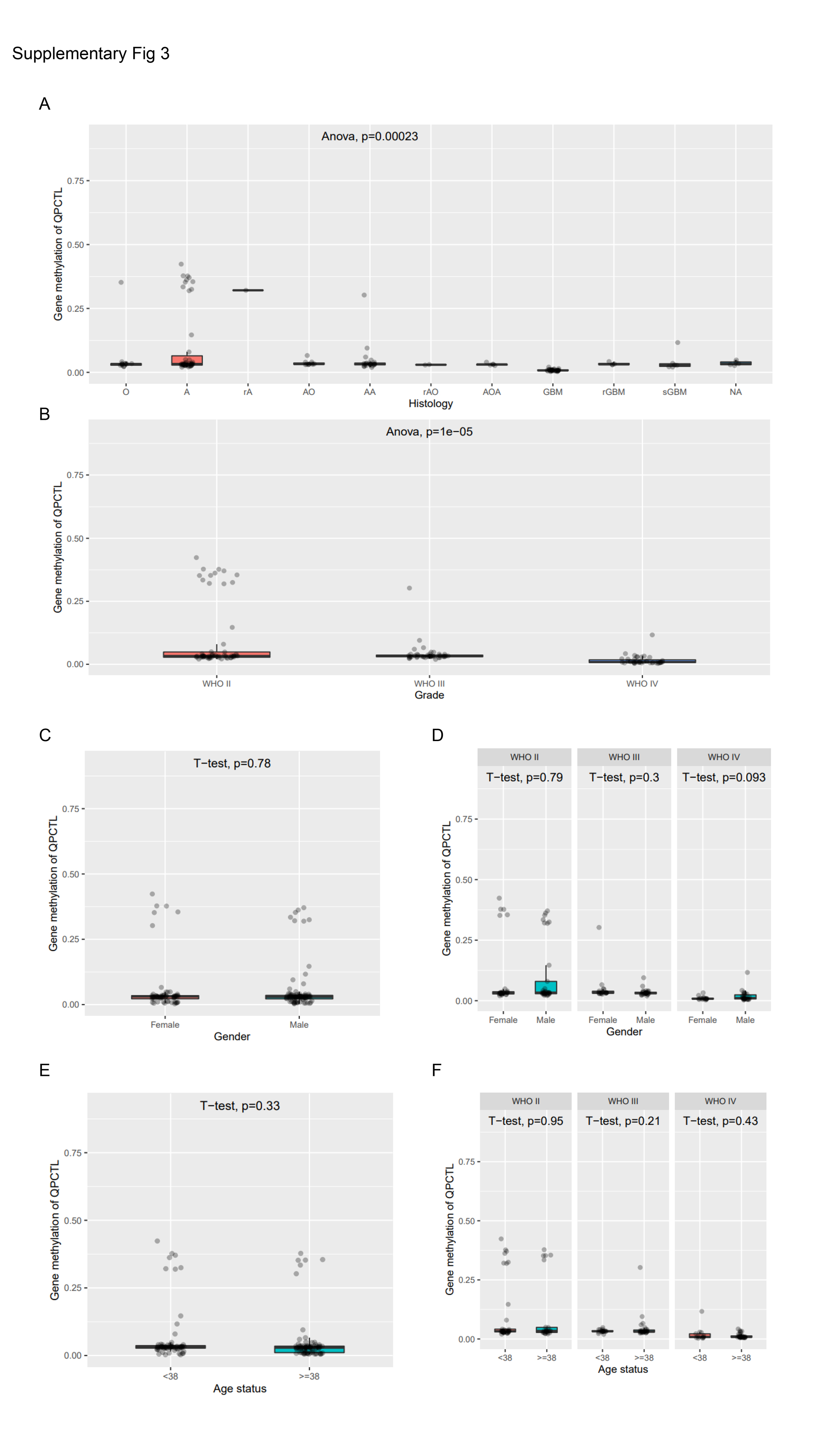

Supplement: Supplementary Figure 3 — The DNA methylation level of QPCTL in the CGGA database. (A) The DNA methylation level of QPCTL in different histology of glioma. (B) The DNA methylation level of QPCTL in different pathological stages of glioma. (C) The DNA methylation level of QPCTL in different genders. (D) The different DNA methylation levels of different genders in different pathological stages. (E) The DNA methylation level of QPCTL in different ages. (F) The different DNA methylation levels of different ages in different pathological stages. [file Image_3.jpeg]

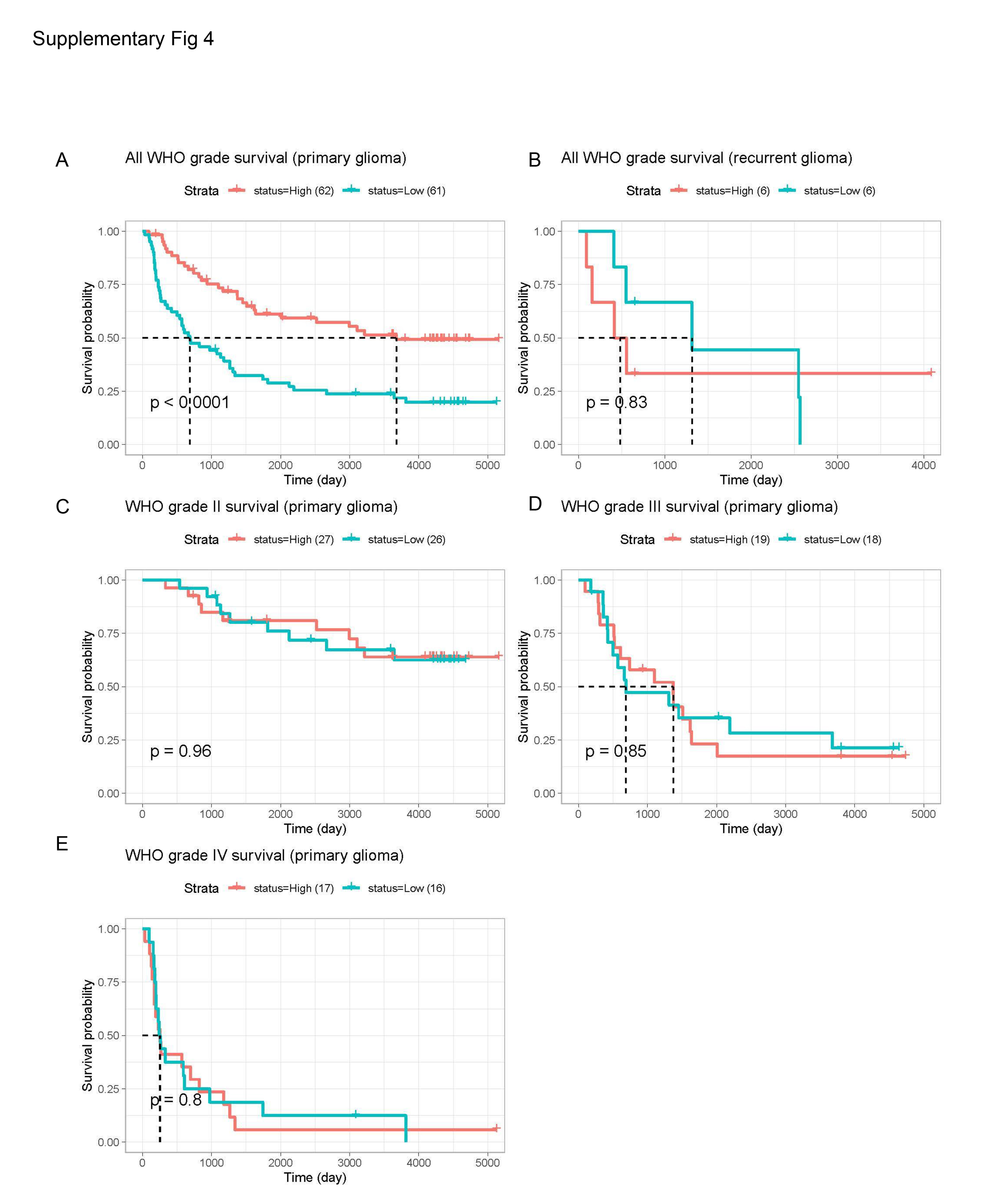

Supplement: Supplementary Table 1 — QPCTL CRISPR screen data from BIOGRID. [file Image_4.jpeg]
